# The effects of fire on ant trophic assemblage and sex allocation

**DOI:** 10.1002/ece3.714

**Published:** 2013-12-06

**Authors:** Stephane Caut, Michael J Jowers, Xavier Arnan, Jessica Pearce-Duvet, Anselm Rodrigo, Xim Cerda, Raphaël R Boulay

**Affiliations:** 1 Estación Biológica de Doñana, Consejo Superior de Investigaciones Científicas (CSIC)Av. Americo Vespucio s/n, 41092, Sevilla, Spain; 2 CREAFCampus UAB, E-08193, Bellaterra, Spain; 3 Departamento de Zoología, Universidad de GranadaCampus Fuente Nueva, 18071, Granada, Spain; 4 Facultat de Biociències, Universitat Autònoma de BarcelonaE- 08193, Bellaterra, Spain; 5 Institut de Recherches sur la Biologie de l'Insecte, CNRS UMR 7261, Université François Rabelais de ToursTours, 37200, France

**Keywords:** Ant assemblage, *Aphaenogaster gibbosa*, reproductive output, sex ratio, stable isotopes, wildfire

## Abstract

Fire plays a key role in ecosystem dynamics worldwide, altering energy flows and species community structure and composition. However, the functional mechanisms underlying these effects are not well understood. Many ground-dwelling animal species can shelter themselves from exposure to heat and therefore rarely suffer direct mortality. However, fire-induced alterations to the environment may change a species' relative trophic level within a food web and its mode of foraging. We assessed how fire could affect ant resource utilization at different scales in a Mediterranean forest. First, we conducted isotopic analyses on entire ant species assemblages and their potential food resources, which included plants and other arthropods, in burned and unburned plots 1 year postfire. Second, we measured the production of males and females by nests of a fire-resilient species, *Aphaenogaster gibbosa,* and analyzed the differences in isotopic values among workers, males, and females to test whether fire constrained resource allocation. We found that, in spite of major modifications in biotic and abiotic conditions, fire had little impact on the relative trophic position of ant species. The studied assemblage was composed of species with a wide array of diets. They ranged from being mostly herbivorous to completely omnivorous, and a given species' trophic level was the same in burned and unburned plots. In *A. gibbosa* nests, sexuals had greater δ^15^N values than workers in both burned and unburned plots, which suggests that the former had a more protein-rich diet than the latter. Fire also appeared to have a major effect on *A. gibbosa* sex allocation: The proportion of nests that produced male brood was greater on burned zones, as was the mean number of males produced per nest with the same reproductive investment**.** Our results show that generalist ants with relatively broad diets maintained a constant trophic position, even following a major disturbance like fire. However, the dramatically reduced production of females on burned zones compared to unburned zones 1 year postfire may result in considerably reduced recruitment of new colonies in the mid to long term, which could yield genetic bottlenecks and founder effects. Our study paves the way for future functional analyses of fire-induced modifications in ant populations and communities.

## Introduction

Wildfires constitute a major disturbance force in forest ecosystems worldwide and cause profound alterations in habitat structure, energy flow, and species community composition (Bengtsson et al. 2000; Moretti et al. 2004, 2010). Although wildfire frequency and intensity are predicted to increase substantially by midcentury (Bradstock 2008; Westerling et al. 2011), their effects on biodiversity and ecosystem functioning are still debated (Gill et al. 1999; Copelan et al. 2002). Numerous studies have shown that the composition of animal communities and/or the abundance of animals therein vary greatly between burned and unburned areas, yet the functional mechanisms responsible for these patterns are still not well understood, particularly in invertebrate communities (Anderson et al. 1989; Gill et al. 1999; Arnan et al. 2006, 2007; Cobb et al. 2007; Gillette et al. 2008; Parr and Andersen 2008; Andersen and Hoffmann 2011).

Fire can affect the arthropod community either through its direct consequences, that is, mortality induced by burning, or its indirect impact on plant ecological succession. Although the fire-induced mortality of epigeic species is high, many ground-dwelling arthropods do survive fire, mostly because the heat does not reach depths greater than 30–40 cm below the surface, not even when ground surface temperature exceeds 400°C (DeBano 2000). On the other hand, several lines of evidence indicate that arthropods may be strongly affected by new, postfire environmental conditions. It appears that the significant amount of vegetative cover generated by early successional plants likely alters the whole trophic web via a cascade effect. For example, many fire-adapted plants, over the course of their germination, flowering, and fruiting, have been shown to attract new guilds of pollinators, herbivores, and seed dispersers (Potts et al. 2001; Moretti et al. 2006). Moreover, the abundance of dead wood and the increase in bare ground are known to favor colonization by xylophagous and detritivorous insects (Moretti and Legg 2009). Fire-induced alterations in the composition and abundance of plants and herbivores may in turn translate into alterations at higher trophic levels, including in the composition and abundance of predators and scavengers. Specialist consumers should be the most affected by postfire habitat alterations, whereas more generalist species, such as omnivores, are expected to adjust their diet progressively in response to the new, changing environment. To date, however, too few studies have adopted a functional approach to investigate the effect of postfire conditions on arthropod trophic interactions and fitness. Ants constitute interesting model organisms for such studies. They dominate many forest ecosystems, both demographically and functionally, and are commonly used as bioindicators in environmental conservation efforts (Majer and Nichols 1998; Andersen and Majer 2004; Graham et al. 2009). Moreover, it has been suggested that many ants nesting in the ground survive fire, but are subsequently positively or negatively affected by changes in resource availability (Neumann 1992; Jackson and Fox 1996; Arnan et al. 2006).

In temperate and Mediterranean ecosystems, studies based on direct observations have shown that ant species span a wide range of trophic levels. They range from being granivores and aphid tenders to predators and scavengers, with omnivores being the most abundant (Hölldobler and Wilson 1990). Nevertheless, ant trophic ecology studies that use direct observations are limited by the fact that only a fraction of the food retrieved by foragers (carried in their mandibles or in their crops) may be assimilated. In addition, food may be unevenly distributed among colony members (queens, workers, larvae [LI], etc.). Thus, stable isotope analysis provides a powerful alternative approach in such circumstances (Kelly 2000; Caut et al. 2009, 2013a). This method is based on the notion that an organism's nitrogen and carbon isotopic ratios (δ^13^C and δ^15^N) provide a record of the resources it has assimilated (see Post 2002; for review). In spite of some notable variability across taxa, tissues, and diets, δ^15^N is typically considered to be enriched by ∼3‰ with respect to the diet and consequently reflects an animal's trophic position (e.g., see review Caut et al. 2009; Martinez del Rio et al. 2009). In contrast, δ^13^C changes less between trophic levels (about +1‰; Post-2002) and is used to distinguish between different sources of carbon, mainly C_3_ and C_4_ plants.

In comparison to their use in other study systems, stable isotopes have not widely been used to analyze ant community trophic structure (Feldhaar et al. 2010), and most studies have generally been focused on invasive species (e.g., Mooney and Tillberg 2005; Tillberg et al. 2007; Wilder et al. 2011). Blüthgen et al. (2003) explored the differences in trophic levels of 50 ant species found in tropical Australia and confirmed the presence of a high proportion of omnivores, along with a few specialized herbivores and predators. They also highlighted the fact that the dominant ant *Oecophylla smaragdina* showed a significant dietary shift when it occurred in mature forest versus early successional forest. More recently, Gibb and Cunningham (2011) compared ant trophic levels in three types of habitat (pastures, revegetated pastures, and remnant woodlands). Although the trophic structure of the entire ant assemblage was conserved across habitat types, the overall trophic level of ant species was lower in revegetated pastures, probably as a consequence of the higher availability of plant sugars, honeydew, and herbivore insect prey.

Another interesting aspect of ant ecology is that resource abundance and quality affect important trade-offs, such as the trade-off between colony growth (production of sterile workers) and reproduction (production of sexuals), as well as the trade-off between male production and queen production. For example, food supplementation in the field has been shown to increase colony queen production in several species of *Formica* (Deslippe and Savolainen 1995; Brown and Keller 2006), *Messor* (Ode and Rissing 2002) and *Myrmica* (Bono and Herbers 2003), which suggests that the production of queens is limited by resource availability. Providing lipid- or protein-rich diets to laboratory colonies has been shown to have contrasting, seemingly species-specific effects on sexual production (e.g., Morales and Heithaus 1998; Backus and Herbers 1992; Herbers and Banschbach 1998; Barroso et al. 2013). Aron et al. (2001) persuasively showed that the addition of maggots to a sugar-based diet increased the production of both males and queens in the Argentine ant *Linepithema humile* in the laboratory, suggesting that sexual LI are more protein limited than workers. This hypothesis was recently supported in *Pogonomyrmex badius*; males and queens were found to have more elevated δ^15^N values, and thus probably higher protein intake, than workers (Smith and Suarez 2010). Based on these previous results, we hypothesized that wildfires, by changing resource availability, may also indirectly affect the way in which colonies allocate resources to growth versus reproduction and, among reproductives, to males versus females.

The aim of this study was to determine the effect of wildfires on resource availability and the utilization of available resources by ants at the community and colony levels. We hypothesized that the major fire-induced modifications in animal and plant communities would secondarily affect the entire food web and ant colony sex allocation. We tested this hypothesis using ant species in a Mediterranean forest that had burned 1 year prior. First, we tested the effect of fire on ant community trophic structure. We began by comparing the isotopic values of ant species when they occurred on burned versus unburned areas of the same forest to determine the effect of fire on species trophic level. Then, we identified the most important ant food resources (invertebrates and plants) and characterized their availability; ant isotopic values were subsequently compared to those of their potential food resources to clarify resource assimilation. Second, we excavated colonies of focal species *Aphaenogaster gibbosa* during its period of sexual production to test whether fire had provoked a modification in resource allocation at the colony level. Finally, we compared the isotopic values among castes to test the relative roles of resource selection versus habitat conditions in caste determination.

## Material and Methods

### Study site

**Figure 1:**
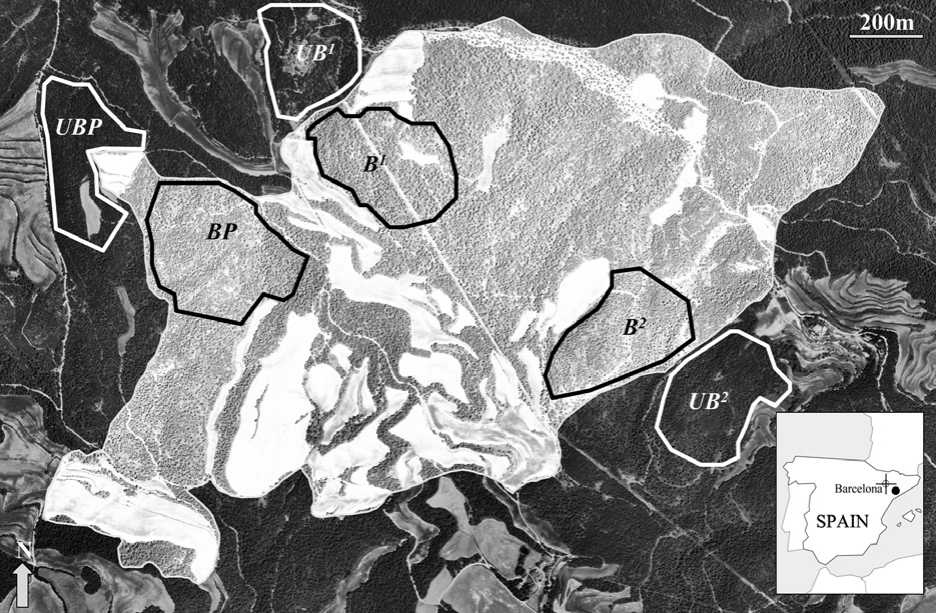
Map of the burned area (light gray) and the different study sites. The areas outlined in black are the burned zone; *BP* contained the burned plots, and the two pairs of additional burned sites are labeled *B*^1^ and *B*^2^. The areas outlined in white are the unburned zone; *UBP* contained the unburned plots (*UBP*), and the two pairs of additional unburned sites are labeled *UB*^1^ and *UB*^2^.

The study was conducted near the village of Salo in northeastern Spain (41°52′N 1°38′E) on areas ranging between 540 m and 620 m in altitude. The climate in this area is typically Mediterranean and tempered by trade winds. The mean daily temperature recorded in the city of Manresa (about 20 km from the study site) over the last 20 years ranges between 4.4°C in January and 23.7°C in July. Annual rainfall averages 585 mm; it is lowest in July and highest in September. The landscape is craggy and formed by a complex patchwork of cultivated fields and pine forests (*Pinus nigra* and *Pinus halepensis*); the forests have a relatively dense understory composed of typical Mediterranean shrubs, including *Rosmarinus officinalis*, *Thymus vulgaris*, *Rhamnus alaternu*s, and *Lavandula latifolia* (DMAH 2005). In June 2009, an accidental crown fire burned 194 ha (Figs. 1 and S1) of woodlands (74%), cultivated fields (24%), and scrubland (2%). All trees (predominantly pine) were almost completely burned. Occasionally some trunks (still standing) remained in the burned ares and provided limited canopy cover. Fallen branches were occasionally present on the ground and were not removed. Photographs are available in the appendix (Fig. S1).

### Sampling

Vegetation and arthropod sampling was conducted in ten 10 × 10 m plots set up in the burned site (*BP*, *n* = 5) and the adjacent unburned site (*UBP*, control, *n* = 5, hereafter called “plots”). The position of the plots was chosen such that they fulfilled all the following conditions: (1) Each plot contained at least one *A. gibbosa* nest in its center; (2) The distance between plots ranged from 50 to 500 m, which guaranteed their independence as sampling units; (3) Before the fire, the burned and unburned plots belonged to the same continuous wooded area and had similar vegetation and abiotic conditions (e.g., slope, aspect, rockiness, orientation); (4) They were sufficiently distant from the fire's edge and from the cultivated fields (∼100 m) to limit edge effects; and (5) No management measure such as the removal of dead wood and/or revegetation was undertaken after the wildfire. The last condition is particularly important and limiting as many burned areas are rapidly submitted to management measures that may have major consequences on the ecosystems on the short term. Data loggers (HOBO®, Onset, Bourne, MA) installed during the month of July 2010 indicated that maximum daily ground temperature was about 6°C higher in the burned site, whereas the minimum relative humidity was about 12% lower (Fig. S1).

#### Plants and soil

Two 0.8 L soil samples were collected from each plot for chemical, granulometric, and isotopic analyses. The plant community of each plot was characterized at the end of June 2010 by visually estimating the relative area occupied by each species. One specimen per species (leaves, flowers, or seeds) per plot was also collected between May and June and kept in a sealed paper envelope for later isotope analyses.

#### Invertebrates

Invertebrates were sampled in late June of 2010 over three consecutive days using seven pitfall traps (20 cm^3^ plastic cups) and two yellow pan traps (20 × 5 cm yellow plastic plates) per plot. Pan traps were used to increase the probability of capturing flying insects. Pitfall and yellow pan traps were partially filled with soapy water. The arthropods collected using both trap types were pooled daily for each plot and stored in 70% alcohol until they could be identified in the laboratory. Ants were identified to the species level, whereas nonant invertebrates were identified to the order or family level. The total insect biomass (wet mass Pesola AG, Baar, Switzerland) was measured using precision *Pesola*® scales accurate to the nearest 0.1 mg.

#### Aphaenogaster gibbosa

The 16 nests of *A. gibbosa* present in the plots (nine in burned plots and seven in unburned plots) were excavated in their entirety in early July 2010, just after invertebrate sampling. The nests were dug up gradually, and all individual ants and brood were carefully collected using a hand-held battery-powered vacuum. For each of these nests, the LI, workers (adults and pupae), and sexuals (adults) were counted and kept in 70% alcohol until isotope analyses could be performed.

To increase our sample size and control for broad-scale spatial variation in sex allocation, we completely excavated 10 nests from the same site in which our plots were located and 16 nests from two additional pairs of adjacent burned and unburned sites (*B*^1^–*UB*^1^ and *B*^2^–*UB*^2^, see Fig. 1); these additional sites were located 500–1000 m from the study plots. As nest excavation was extremely time consuming and difficult in our study area (requiring 2–3 h per nest), we also excavated partial nests in the burned and unburned zones (burned zone = 3 sites *BP*–*B*^1^–*B*^2^ and unburned zone = 3 sites *UBP*–*UB*^1^–*UB*^2^, Fig. 1): We collected sexuals by lifting numerous flat stones and rapidly vacuuming up all the sexuals located beneath (15 and 12 nests in the burned and unburned zones, respectively). From among these additional nests, 21 nests were sampled to analyze the spatial variation in worker isotopic values present across a larger area. In total, isotope analyses were conducted on 16 nests to compare burned and unburned plots and 37 nests to find worker spatial variation in burned and unburned zones. Sex ratio analyses were conducted on 42 complete nests and 27 partial nests in burned and unburned zones.

Moreover, temporal variation in worker isotopic values was assessed by using workers captured during pitfall trapping conducted in July 2009 in the burned and unburned zones (same protocol as above). Thus, we could compare the isotopic values of workers from 2010 with those of workers collected in 2009, just 1 month after the fire.

Numerical and investment sex ratios (*nSR* and *iSR*, respectively) were estimated for complete (^T^, *n* = 42) and partial (^P^, *n* = 27) *A. gibbosa* nests collected in the burned and unburned zones. *nSR* was the proportion of female sexuals (females/males + females). *iSR* was calculated by determining the total female dry mass (number of females multiplied by mean individual female biomass) and dividing it by the total sexual dry mass (number of males and females multiplied by their respective mean individual biomass). For completely excavated nests for which we calculated *nSR*^*T*^ and *iSR*^*T*^, we also calculated the percentage of reproductive biomass (*R*_*B*_ = biomass of adult sexuals/(biomass of adult sexuals + biomass of workers) × 100), male biomass (*M*_*B*_ = biomass of males/(biomass of adult sexuals + biomass of workers) × 100), and female biomass (*F*_*B*_ = biomass of females/(biomass of adult sexuals + biomass of workers) × 100). Worker, male, and female individual biomass was estimated from samples of 12–20 individuals collected from four different nests in burned and unburned zones. Individuals were first dried for 24 h in an oven at 55°C and weighed to the nearest 0.01 mg.

### Isotopic analysis and soil chemistry

All samples of invertebrates and plants were dried at 60°C for 48 h, ground to a fine powder, weighed in tin capsules, and stored in a desiccator until isotope analyses were performed. In order to reduce the contamination of ant tissues by recently ingested food contained in the stomach, only the thorax and legs were analyzed. In our analysis of the LI, we used only the LI instar to limit the possibility of undigested food in the intestine. Individual measurements were possible in only a few cases (i.e., ant queens). Most often, between two and 30 individuals were pooled per plot, species, and caste in order to obtain sufficient material (0.5 mg dry mass) for accurate isotope ratio determination.

Isotopic analyses were performed using an Optima® mass spectrometer (Micromass, U.K.) coupled to a C–N–S elemental analyzer (Carlo Erba, Italy). Ratios are presented as δ values (‰), which are expressed relative to the vPDB (Vienna Peedee Belemnite) standard for carbon and atmospheric N_2_ for nitrogen. Stable C and N isotope ratios are expressed as: δ^13^C or δ^15^N = [(*R*_sample_/*R*_standard_)−1] × 1000, where *R* is ^13^C/^12^C or ^15^N/^14^N for δ^13^C or δ^15^N, respectively. Reference materials were IAEA-CH-6 (−10.4‰) for δ^13^C and IAEA-N1 (+0.4‰) for δ^15^N. One hundred replicate assays of internal laboratory standards indicate maximum measurement errors (SD) of ±0.2‰ and ±0.15‰ for stable carbon and nitrogen isotopes, respectively. The C/N ratio was calculated as the total percentage of carbon divided by the total percentage of nitrogen.

Soil chemistry, pH, and granulometry were analyzed at the IRNASE (Instituto de Recursos Naturales de Sevilla – CSIC). Phosphorus was measured using the Bray–Kurtz method. Soil granulometry was estimated using the Bouyoucos method.

### Statistical analysis

#### Ant community

Ant species abundance in burned and unburned plots was compared by fitting a generalized mixed-effect model (hereafter GLMM) using the lme4 library (Bates et al. 2011) in R (R Development Core Team 2008). Fixed effects were fire, ant taxa, and their interaction, whereas plot was included as a random effect. The model was fitted with the Poisson distribution and the log link function. A fixed effect was determined to be significant if its removal from the model resulted in a significant reduction in the Akaike information criterion using the Chi-square test. Differences between fixed effect levels were assessed using the *contr.treatment* function, which performs contrast analyses that compare each factor level to a control. Total insect biomass in burned and unburned plots was compared by means of Kruskal–Wallis (hereafter KW) nonparametric tests.

#### Sex allocation

Linear models (LM) were fitted to compare the number and total biomass of workers from *A. gibbosa* nests collected in burned and unburned zones. The distribution of males and females among nests was analyzed by conducting logistic regression on a subset of nests containing at least five sexuals (the presence of males was the response variable, whereas the presence of females was the predictor). Linear mixed models (LMM) were used to compare the number of males and females, *n*SR^T^, *i*SR^T^, *R*_*B*_, *M*_*B*_, and *F*_*B*_ in complete nests in burned and unburned zones. In the analyses, site identity and the number of adult workers were included as random variables; adult worker biomass was a covariable. For partial nests, we could only compare *n*SR^P^.

#### Isotopic trophic interactions

Plant δ^15^N and δ^13^C isotopic values were compared between burned and unburned plots by fitting two LMMs (plot identity was included as a random effect). We repeated the same analysis a second time, using only plant species previously identified as major food resources in a traditional diet study of *A. gibbosa* performed in the same area (*Aphyllantes monspeliensis*, *Fumana ericoides*, *Lithospermum fruticosum*, *Ononis minutissima*, *P. nigra*, *Reseda phyteuma*, and *R. officinalis*; Lazaro-Gonzalez et al. 2013). Differences in soil δ^15^N and δ^13^C between burned and unburned plots were tested using KW nonparametric tests. Differences in invertebrate and ant δ^15^N and δ^13^C values were analyzed by fitting LMMs in which fire, taxa (either invertebrate taxa or ant species), and their interaction were included as fixed effects; plot identity was included as a random effect. LM were also fitted to test spatial differences in the isotopic values of *A. gibbosa* workers in burned and unburned zones (site identity was included as random variable). The fire effect was nested within the pairs of sites. Temporal variation in samples collected 1 month and 1 year after the fire in burned and unburned zones was tested using LM (site identity was included as random variable).

The SIBER (Stable Isotope Bayesian Ellipses in R; Jackson et al. 2011) procedure from the SIAR package (version 4.1.3) was employed to compare ecosystem (all plants and invertebrates) and ant assemblage (all ant species) isotopic niche widths between the burned and unburned plots. Our sample size was too small to conduct this analysis at the ant species level. The SIBER procedure generates standard ellipse areas (SEA_B_), which are bivariate equivalents of standard deviation in univariate analysis. As per Jackson et al. (2011), we graphically expressed SEA_B_ using a corrected SEA_B_ value to minimize bias across the ranges of sample size for each population. We also calculated community metrics for the ecosystem (plants and nine invertebrate groups) and the ant assemblage (eight groups of species) in the burned and unburned plots to describe the arrangement of taxonomic/functional groups as part of the larger community (Layman et al. 2007a,b; Jackson et al. 2011): The six classical Layman's metrics: *NR* reflects the trophic length of the community; *CR* reflects niche diversity at the base of the food web; *TA* is the total area of the convex hull encompassed by all species in biplot space, which gives an indication of niche width; *CD* is the mean distance to the centroid, which provides a measure of the average degree of trophic diversity within food web, but is also a function of the degree of species spacing; *MNND* is mean nearest neighbor distance, which provides an estimate of the overall density of species packing; and *SDNND* is the standard deviation of nearest neighbor distance, which is a measure of the evenness of species packing in biplot space.

Finally, LMMs were used to test the effect of life stage (larva, pupal worker, adult worker, male, female, or queen) and fire on δ^13^C, δ^15^N, and the C/N ratio (plot identity, as well as nest nested within plot, were included as random effects). We used the Tukey test to conduct multiple comparisons of the means of the different castes (adult workers and pupal workers; males, females, and queens). The relationship between δ^13^C and the C/N ratio was tested using a general regression model.

All estimates are means ± SE unless otherwise specified.

## Results

### Fire-induced modifications to soil, plants, and arthropods

**Figure 2:**
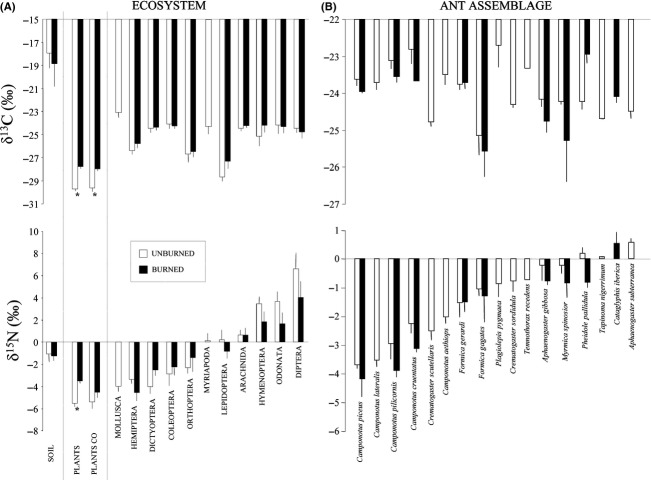
Mean (+SE) δ^13^C and δ^15^N values of the five plots: (A) the ecosystem: soil, plant (Plants = all plants; Plants CO = plants consumed by ants), and invertebrate communities (by order for Arthropoda) and (B) the ant assemblage: different species in the community (worker isotopic values). Values for burned and unburned plots are represented by black and white bars, respectively. Asterisks indicate significant differences between burned and unburned plots for specific plant groups (Kruskal–Wallis nonparametric comparison, *P < 0.05). Invertebrate groups and ant species have been ordered by consumer type (plant to animal diet).

Fire resulted in important modifications in biotic and abiotic conditions. The soil was composed of a higher proportion of coarse sand in the burned plots, but soil chemical and isotopic composition were not significantly different between burned and unburned plots (Figs. 2A and Table S1). Whereas the unburned plots were covered by a dense canopy of *P. nigra* (83%), the burned plots were characterized by a higher proportion of bare ground (∼50%, Figs. S1, S2). Of the total of 35 herb and shrub species observed, 15 and 11 species were exclusively found in unburned and burned plots, respectively (Fig. S2). This difference was unlikely to have existed before the fire as the entire study area was very homogenous. The understory in the unburned plots was dominated by various species of Poaceae and Bryophyta; they were completely absent from the burned plots, in which understory shrubs such as *Arbutus unedo, Brachypodium retusum*, and *Quercus cerrioides* were abundant. Plants collected in the burned plots had significantly higher δ^15^N and δ^13^C values than those collected in the unburned plots (Fig. 2A: δ^15^N: *F*_1,140_ = 44.39, *P* < 0.001; and δ^13^C: *F*_1,140_ = 54.24, *P* < 0.001). However, when we restricted our analysis to those species known to be retrieved by *A. gibbosa* (Lazaro-Gonzalez et al. 2013), δ^13^C remained significantly higher (Fig 2A, LMM: *F*_1,29_ = 8.86, *P* = 0.006) whereas δ^15^N did not (LMM: *F*_1,29_ = 1.72, *P* = 0.200).

A total of 1367 and 1604 invertebrates were captured (pitfall + yellow pan traps) in the burned and unburned plots, respectively; the majority were arachnids and insects (Fig. S3). The effect of fire on invertebrate abundance varied according to taxa, as evidenced by the significant interaction between taxa and fire (LM, Taxa × Fire: χ^2^_12_ = 1451.2, *P* < 0.001, Fig. S3). Hence, the abundance of dipterans, hemipterans, hymenopterans, myriapods, molluscans, and odonatans was significantly greater in unburned than burned plots (Fig. S3). Total insect biomass was significantly greater in burned than unburned plots (1.27 ± 0.16 g vs. 0.45 ± 0.23 g, respectively; KW test: *H*_1,10_ = 6.82, *P* = 0.009), probably as a consequence of the higher number of coleopterans captured in the former.

The main invertebrate taxa had significantly different δ^15^N and δ^13^C values (Taxa: *F*_8,158_ = 13.50, *P* < 0.001; and *F*_8,158_ = 12.42, *P* < 0.001, respectively; Fig. 2A) that matched their known dietary regimes; scavengers and predators (e.g., dipterans and odonatans) had higher δ^15^N values than phytophagous invertebrates (e.g., molluscs and lepidopterans). These differences were independent of fire (LM: δ^13^C: Fire: *F*_1,8_ = 0.46, *P* = 0.516, and Fire × Taxa: *F*_8,158_ = 0.54, *P* = 0.825; δ^15^N: Fire: *F*_1,8_ = 0.56, *P* = 0.475, and Fire × Taxa: *F*_8,158_ = 0.64, *P* = 0.745, Fig. 2A).

**Figure 3:**
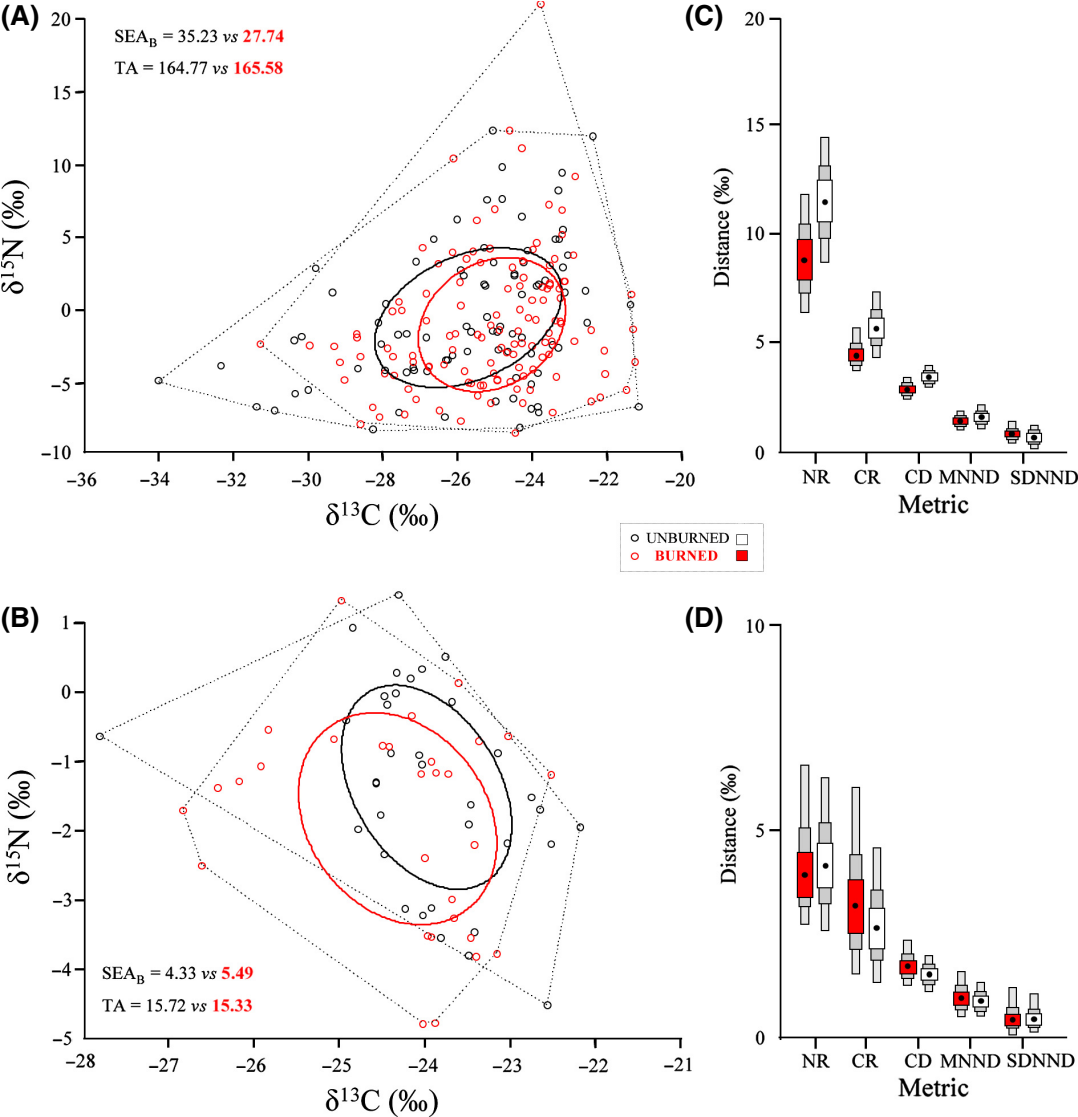
δ^13^C and δ^15^N biplots of (A) the entire ecosystem and (B) the ant assemblage in burned (red circles) and unburned (white circles) plots. Convex hulls of total niche width (as per Layman et al. 2007a) are depicted using dashed lines (TA). The standard ellipse area (SEA_B_) representations of isotopic niches, that is, the bivariate equivalent of SD in univariate analysis (as per Jackson et al. 2011), are depicted for burned (solid red lines) and unburned (solid black lines) plots. Corresponding niche/community Layman metrics (*NR, CR, CD, MNND,* and *SDNND*, see details in methods, as per Jackson et al. 2011) are shown for (C) the entire ecosystem and (D) the ant assemblage in burned (red bars) and unburned (white bars) plots. The 5th to 95th percentile range of the distribution is plotted, and the black dots indicate the mode.

Niche width was not significantly different between burned and unburned plots (SEA_B_ = 35.23 vs. 27.74, respectively, *P* = 0.549, Fig. 3A), given the 86% overlap in SEA_B_ between the two ellipses. Moreover, when we compared community-wide metrics between burned and unburned plots, we found that they were similar (Fig. 3C).

### Effects of fire on ant trophic position

**Figure 4:**
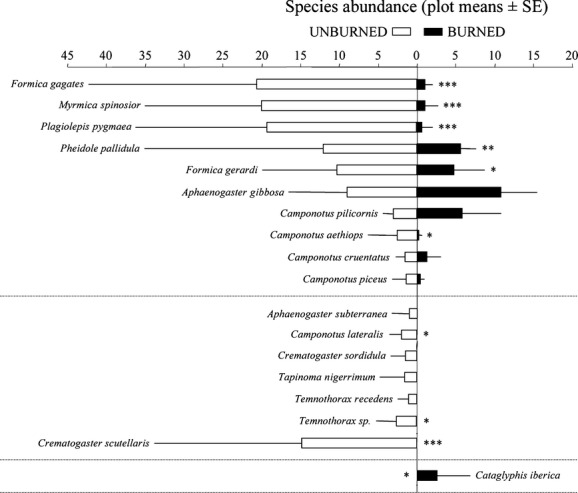
Mean ant species abundance (number of individuals) for burned (black bar) and unburned (white bar) plots (means ± SE, *n* = 5). The mean number of individuals of each group collected within each plot was used as the basis of this comparison. Asterisks indicate significant differences between burned and unburned plots for specific species (contrast analyses, *P < 0.05; **P < 0.01; ***P < 0.001).

Ants represented the most abundant family among the hymenopterans captured at both areas. The effect of fire on ant abundance differed significantly across species (GLMM, Ant Species × Fire: χ^2^_17_ = 204.5, *P* < 0.001). Nine species found in unburned plots were significantly less abundant in burned plots. Among them, the tree-dwelling ant *Crematogaster scutellaris* was most notably absent, as were *Formica gagates*, *Myrmica spinosior*, and *Plagiolepis pygmaea*. One species, the thermophilic ant *Cataglyphis iberica*, was significantly more abundant in burned than unburned plots. The abundance of the remaining eight species, including *A. gibbosa*, was not significantly affected by fire (Fig. 4).

Ant δ^15^N and δ^13^C also varied across species (LM: Species: *F*_7,41_ = 19.88, *P* < 0.001 and *F*_7,41_ = 7.77, *P* < 0.001, respectively). In particular, δ^15^N values revealed a wide spectrum of trophic niches (range: −4.2–0.6‰), from nectar-feeding species with low δ^15^N (e.g., *Camponotus;* Fig. 2B) to species feeding mostly on animal tissues with elevated δ^15^N (e.g., *C. iberica* and *A. subterranea*). Workers of *A. gibbosa* had an intermediate δ^15^N, which is typical for an omnivorous species that relies both on insect prey and plant-derived food. The range of variation in δ^13^C was lower (−25.6 to −22.7‰) because only C_3_ plants were present in our study area. δ^13^C values did not differ significantly between burned and unburned plots (LM: Fire: δ^13^C, *F*_1,8_ = 3.33, *P* = 0.106 and δ^15^N: *F*_1,8_ = 2.39, *P* = 0.160). Moreover, interspecific differences were independent from fire (Fire x Species: δ^13^C, *F*_7,41_ = 1.08, *P* = 0.397 and δ^15^N: *F*_7,41_ = 0.40, *P* = 0.894, Fig. 2B). The relative size of Bayesian ellipses among ant assemblages shows that the effect of fire on niche width was not significant (Fig. 3B, SEA_B_ = 5.49 and 4.33 for burned and unburned plots, respectively, 75% of overlap, *P* = 0.808). Moreover, the community-wide metrics were all very similar between burned and unburned plots (Fig. 3D).

The addition to the analysis of workers collected from 21 nests in the wider area outside the plots confirmed the lack of a significant effect of fire on *A. gibbosa* isotopic values (LM: δ^15^N, *F*_1,31_ < 0.01, *P* = 0.943; δ^13^C, *F*_1,31_ = 0.31, *P* = 0.583). Moreover, the carbon and nitrogen isotopic values of workers collected from burned and unburned zones 1 year after the fire did not differ significantly from those collected from the same zones just a month after the fire (Fire: δ^15^N, *F*_1,34_ = 2.69, *P* = 0.110, δ^13^C, *F*_1,34_ = 0.87, *P* = 0.358; Time: δ^15^N, *F*_1,34_ = 1.06, *P* = 0.310, δ^13^C, *F*_1,34_ = 0.92, *P* = 0.345).

### Effects of fire on *aphaenogaster gibbosa* reproductive output

**Figure 5:**
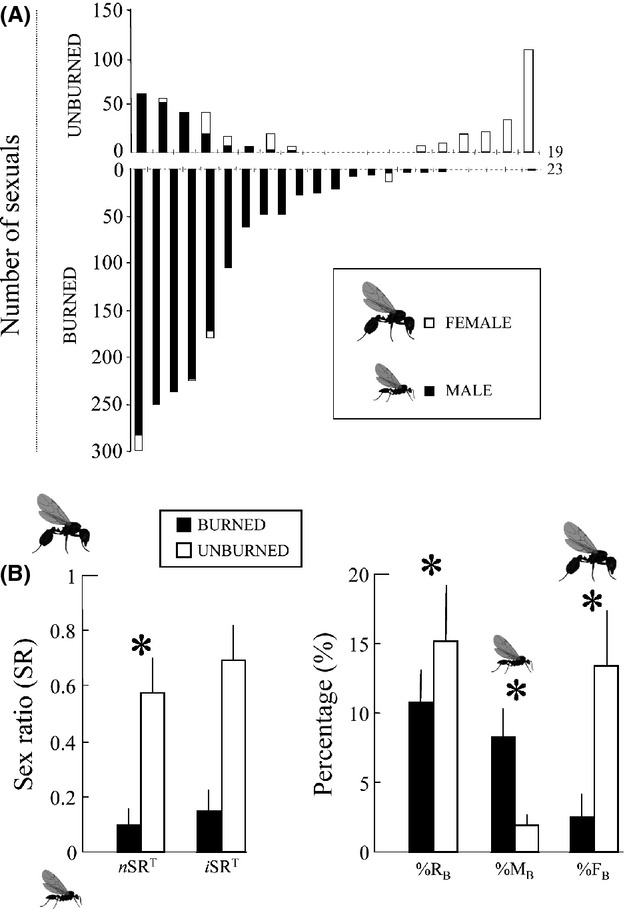
(A) Number of sexuals (black for males and white for females) in the complete nests excavated in burned and unburned zones. Each bar represents one nest (burned, *n* = 23; unburned, *n* = 19). We ordered the nests from highest to lowest according to the number of males they contained. (B) Effect of fire on the numerical sex ratio (*n*SR^T^) and investment sex ratio (*i*SR^T^) as well as on the percentage of reproductive biomass (%*R*_*B*_), male biomass (%*M*_*B*_), and female biomass (%*F*_*B*_) of completely excavated nests. Asterisks indicate significant differences between burned and unburned zones.

A total of 23 and 19 complete nests of *A. gibbosa* were excavated from burned and unburned zones, respectively. Worker number and biomass per nest did not differ significantly between burned and unburned zones (LM: worker number: 415 ± 51 vs. 393±4, *F*_1,40_ = 0.11, *P* = 0.742; worker biomass: 108.55 ± 9.65 g vs. 113.35 ± 8.75 g, *F*_1,40_ = 0.01, *P* = 0.940, respectively). Nineteen (83%) and 14 (73%) nests contained adult sexuals in burned and unburned zones, respectively. Overall, sexual production was split between nests such that those containing females were less likely to contain males (Fig. 5A; GLM: Deviance = 9.37, df = 1, *P* = 0.002). Among nests that produced sexuals, the number of males was significantly greater in burned than unburned zones (Fig. 5A; 80 ± 22 vs. 14 ± 6; KW test: *H*_1,33_ = 6.95, *P* = 0.008), whereas the trend was the opposite for females (2 ± 1 vs. 19 ± 8; KW test: *H*_1,33_ = 10.69, *P* = 0.001). As a consequence, *n*SR^T^ was significantly more male biased for burned than unburned zones (burned *n*SR^T^ = 0.09 ± 0.06 and unburned *n*SR^T^ = 0.57 ± 0.13, Fig. 5B; LMM test: *F*_1,30_ = 19.08, *P* < 0.001). The same trend was seen in partially excavated nests (burned *n*SR^P^ = 0.17 ± 0.09 and unburned *n*SR^P^ = 0.61 ± 0.11; LMM test: *F*_1,19_ = 10.84, *P* = 0.006). Male and female individual dry mass did not differ between burned and unburned zones (male = 0.499 ± 0.155 vs. 0.545 ± 0.123 mg, KW test: *H*_1,40_ = 1.22, *P* = 0.341, gyne = 5.593 ± 0.544 vs. 6.062 ± 0.208 mg, KW test: *H*_1,35_ = 0.03, *P* = 0.854). Therefore, *i*SR^T^ values were estimated using the overall mean male and female individual dry mass, without taking fire into account. Even after accounting for the large difference between male and female dry mass, *i*SR^T^ was still significantly more male biased in burned zones than in unburned zones (Fig. 5B). However, this trend was not significantly different (LMM test: *F*_1,30_ = 23.17, *P* = 0.111) because of the effect of adult worker biomass (LMM test: *F*_1,30_ = 24.92, *P* < 0.001). Finally, the relative biomass of adult sexuals (*R*_*B*_) differed significantly between burned and unburned zones (Fig. 5B; LMM test: *F*_1,30_ = 3.66, *P* < 0.001). The relative biomass of males (*M*_*B*_) and sexual females (*F*_*B*_) was also significantly different; it was higher for males and lower for females in burned zones (LMM test: *F*_1,30_ = 17.68, *P* = 0.011 and *F*_1,30_ = 8.07, *P* < 0.001, respectively, Fig. 5B).

**Figure 6:**
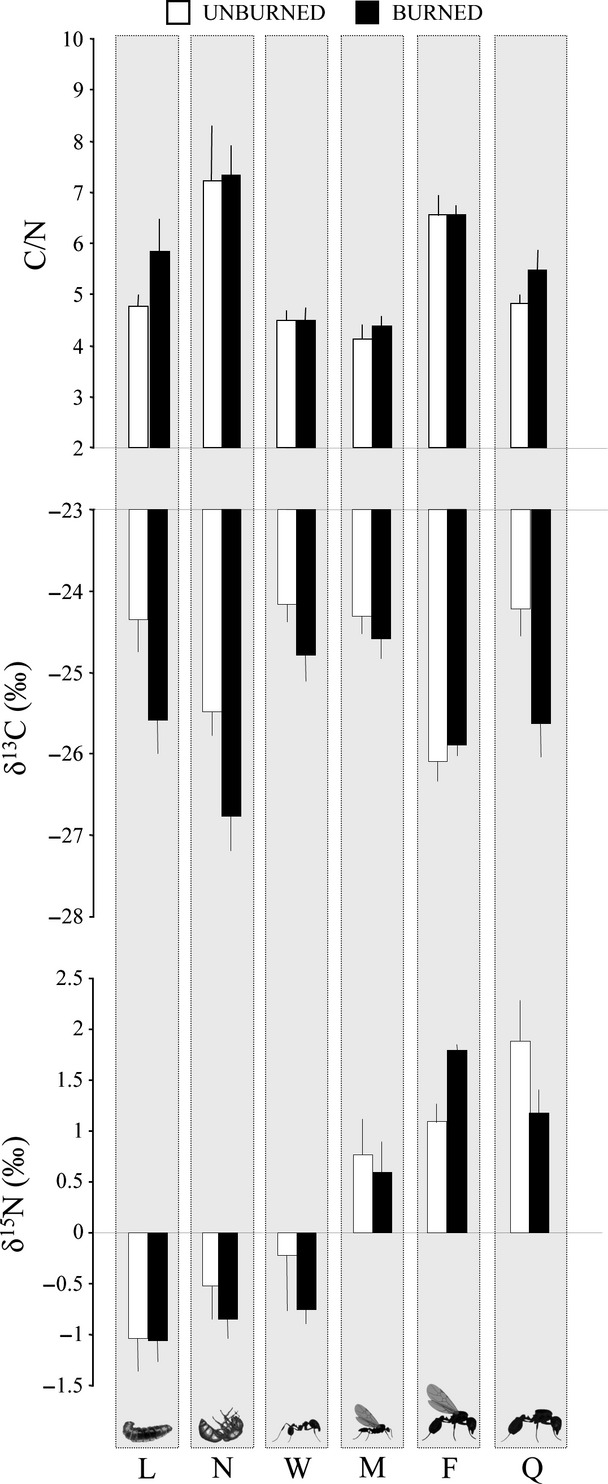
Mean (+SE) C/N ratios, δ^13^C values, and δ^15^N values of the different *A. gibbosa* life stages (in the following order: L, larva; P, pupa; W, worker; M, male; F, female; and Q, queen) for burned (black bars) and unburned (white bars) plots.

Within *A. gibbosa* nests, there was a significant difference in the isotopic values of the different life stages (LMM: δ^15^N – *F*_5,57_ = 17.70, *P* < 0.001 and δ^13^C – *F*_5,57_ = 5.94, *P* < 0.001, Fig. 6). The difference in carbon was likely due to a significant difference in the C/N ratio, which is a proxy for lipid content, among castes (LMM: C/N, *F*_5,57_ = 8.12, *P* < 0.001). The C/N ratio was significantly negatively correlated with δ^13^C (*F*_1,67_ = 83.49, *P* < 0.001, *R*^2^ = 0.55; Fig. 6). This relationship is attributable to the fact that lipids are often depleted in ^13^C relative to other animal tissues; lipid presence can thus complicate the trophic interpretation (Post et al. 2007). Unfortunately, we did not have a sufficient number of samples to perform delipidation. However, the multiple comparisons of means showed that only worker isotopic values (adults and pupae) differed significantly from those of sexuals (Tukey comparisons *P* < 0.01). δ^15^N varied from −1.1 to 1.7‰, with an δ^15^N gradient of LI < pupae < workers < sexuals (males < females and queens; Fig. 6). Fire did not have an impact on these differences (LMM: Fire: *F*_1,57_ = 3.24, *P* = 0.077; and Fire × Stage: *F*_4,57_ = 0.33, *P* = 0.857, Fig. 6).

## Discussion

Although fire is known to constitute one of the most important natural disturbances in forest ecosystems, its effect on the trophic interactions of animal consumers is not well understood. Our study is the first to analyze fire's impact on ants, both at the community and species levels. Indeed, our goal was not to conduct a thorough study of the entire invertebrate community, but rather to focus on the trophic ecology of ants and its potential implications for sex allocation when the habitat is altered by fire. Thus, in order to gain a better understanding of this system, it was necessary to study the whole ecosystem beforehand, a process that included characterizing differences in prey availability in burned and unburned areas. However, the results show that, 1 year postfire, the fire had had little impact on invertebrate communities and the relative trophic positions of ants in spite of its major modifications in biotic and abiotic conditions. However, fire resistance and resilience are closely tied to the fire history of the ecosystem and specific taxa (Moretti et al. 2006). The direct effects of these environmental changes on the invertebrate community are not very clear; they can be positive (Villa-castillo and Wagner 2002), negative (Apigian et al. 2006), or of short-term relevance (Baker et al. 2004). Thus, our finding of trophic resilience could clarify the general absence of modification in invertebrate communities observed more than a year after fire (Bailey and Whitham 2002; Baker et al. 2004; Benson et al. 2007; Taber et al. 2008; Garcia-Dominguez et al. 2010). Therefore, these data suggest that a similar trend could be found on a longer timescale, such as 2–3 years. Parr et al. (2004) confirmed that ant assemblages are highly resilient to fire and found a significant difference between recently burned plots (4–5 months postfire) and unburned plots, but not between less recently burned plots (8–16 months postfire) and unburned plots. They concluded that, only 8 months after a fire, the assemblage had returned to its prefire state. Thus, in our study, even though δ^15^N differed significantly between species and between individuals within nests of *A. gibbosa*, these differences were present irrespective of fire occurrence. However, fire had a major impact on resource allocation in *A. gibbosa* nests; in burned zones, both the proportion of nests that produced male brood increased, as did the mean number of males produced per nest with the same reproductive investment (%R_B_).

### Fire's effects on trophic cascades

The most conspicuous effect of the fire was, obviously, the massive incineration of *P. nigra* trees and the understory. One year after the fire, more than 40% of the ground surface was bare. Furthermore, the majority of plants species growing in the burned plots were completely different from those present in the unburned plots. This important alteration in the plant assemblage was accompanied by a decrease in insect taxa diversity. In contrast, insect biomass increased significantly as a consequence of the high abundance of large coleopterans, which were likely attracted to the burned sites by decomposing wood (Moretti et al. 2004). The abundance of various ant species decreased significantly after the fire. Those that were the most negatively affected were tree-dwelling species like *C. scutellaris* and those nesting in the soil surface like *P. pygmaea*. Inversely, species that nested deeper in the ground like *A. gibbosa* (about 30–40 cm) were better protected from the heat and were as abundant in burned as in unburned plots. One thermophilic species, *C. iberica,* was particularly abundant in the burned plots. The short time interval between the fire and our pitfall sampling (1 year) meant that the area was unlikely to have been recolonized by all ant species. It is reasonable to suppose that the extensive bare ground in the burned plots would have allowed workers from nests located at their borders to forage far into their interiors (>50 m); nevertheless, such incursions did not occur in the unburned plots.

In spite of these major fire-induced changes in plant and arthropod community composition, the analyses of stable isotopes did not provide evidence that fire affected the trophic structure of invertebrate resources and, more precisely, the ant trophic assemblage (e.g., isotopic values and niche width SEA_B_). Indeed, food-web structure was not affected by fire; the total extent of spacing within the δ^13^C–δ^15^N biplot space (i.e., community-wide measures of trophic diversity represented by *NR*, *CR*, *TA*, and *CD*) and the positioning of groups relative to each other within niche space (represented by *MNND* and *SDNND* metrics, Layman et al. 2007a,b) were similar in burned and unburned plots. This comparison was possible because soil samples indicated that isotopic baselines did not differ between burned and unburned plots. As in Gibb and Cunningham (2011), plants were not the most suitable indicators of the habitats' isotopic baselines as species composition was very different between habitat types. Hence, the high plant δ^15^N observed in burned plots was a consequence of a modification in plant species composition. In this context, it is worth noting that the abundance of Fabaceae species (typically nitrogen fixers) was clearly higher in burned than unburned plots (see Fig. S2). However, when we restricted our analysis to the major plant resources consumed by ants (especially *A. gibbosa*, Lazaro-Gonzalez et al. 2013), we did not find a significant difference in δ^15^N. Thus, we could assume that the isotopic baseline was not modified by fire. Moreover, there are many winged invertebrate species that probably move across and feed in both areas.

The comparison of ant isotopic values with those of other arthropods indicated that the ant community spanned the whole dietary range. Indeed, if we estimated the ants' trophic enrichment in nitrogen (+3‰ by Feldhaar et al. 2010), we observed species with δ^15^N values as low as those of strict vegetarians (e.g., δ^15^N plants + 3‰; *Camponotus ˜* molluscs) and as high as those of carnivores (e.g., δ^15^N coleopterans + 3‰; *Aphaenogaster subterranea* and *C. iberica ˜* arachnids, Fig. 2). Several species, such as *A. gibbosa*, were located between these extremes and thus probably had mixed diets composed of dead insects and seeds (*Aphaenogaster*) and/or nectar (*Formica*). A similar pattern has been reported in other recent studies, confirming the power of stable isotopes to identify species trophic level (Blüthgen et al. 2003; Ottonetti et al. 2008; Gibb and Cunningham 2011). Previous traditional dietary analyses based on direct observations have revealed important differences between species (e.g., Cerda et al. 1998). However, the relative importance of plant-derived sugars in their diets was difficult to assess because these liquids are generally transported in the forager's crop. Moreover, direct observations do not allow finer distinctions in the trophic levels of omnivorous species (Mooney and Tillberg 2005).

A relative inflexibility in ant trophic levels across different degrees of habitat perturbation has already been reported in previous studies (Blüthgen et al. 2003; Gibb and Cunningham 2011). A notable exception has been seen in the case of the dominant arboreal ant *O. Smaragdina*, which shifted to more proteinaceous resources in regenerating Australian forests (Blüthgen et al. 2003). Gibb and Cunningham (2011) suggested that this pattern of conserved diets could support the hypothesis that past and current interspecific competition constrains ant diet. It is also possible that each species has specific needs. Hence, even the most omnivorous species may not be as opportunistic as it seems at first glance. Further studies are required to test this hypothesis.

### Fire's effects on *A. gibbosa* sex allocation

One of the main findings of our study is the major shift in sex ratio toward the production of males following fire. Although temperature can modify the annual cycles of ant species (Hölldobler and Wilson 1990), we found that the frequency of occurrence of sexuals in burned and unburned zones was very similar. Furthermore, sexual production in *A. gibbosa* occurs over a very short time period, and thus we can hypothesize that *A. gibbosa*'s cycle was at the same point in both burned and unburned zones. Our intention in excavating nest during this time period was to clarify colony trophic ecology. To our knowledge, this is the first study to examine the effect of fire on resources and sex allocation in ants. Sorvari and Hakkarainen (2007) investigated the effect of spruce forest logging on *Formica aquilonia* sex allocation and found, in contrast to our study, that logging provoked a shift toward female production.

Several studies have shown that food supplementation in the field can bias the sex ratio toward the production of females (Deslippe and Savolainen 1995; Morales and Heithaus 1998; Ode and Rissing 2002; Bono and Herbers 2003; Brown and Keller 2006) or males (Backus and Herbers 1992; Herbers and Banschbach 1998), lead to an increase in reproductive allocation (Aron et al. 1994), or have a limited effect (Caut et al. 2013b). However, in this study, isotope analyses suggest that the observed effect of fire on sex allocation was unlikely to be due to a qualitative shift in resource availability. Moreover, Lázaro-González et al. (2013), as a result of foraging observations, found no significant difference in the quantity of food and the proportion of animal and plant items retrieved between burned and unburned areas from the same study zones of this study. However, they did observe that different plant items were collected depending on their availability. Indeed, the complementary use of traditional observations and isotopic dietary analysis in ants has confirmed that nitrogen isotopic values shift when there is a small modification in the diet (Caut et al. 2013a). In our study, fire had no effect on overall ant isotopic values across time; no difference was observed between values obtained 1 month and 1 year after fire. Thus, male and female δ^15^N values were not significantly different in either area, which indicates that both sexes had qualitatively similar diets. However, a reduced amount of resources may have prevented nests from rearing females. The higher δ^15^N values of sexuals compared to workers in both burned and unburned zones suggests that the latter fed on a less protein-rich diet, confirming the recent results of Smith and Suarez (2010). Indeed, the δ^15^N values of sexuals (following correction for trophic enrichment; Feldhaar et al. 2010) suggest they consume more arthropods (e.g., ∼−2‰ Coleopteran), whereas the more negative values of workers suggest they consume more plants (∼−4‰). This finding supports the hypothesis that male and female sexuals may need to consume more protein than workers in order to develop the strong thoracic musculature that activates their wings (Tillberg et al. 2006; Menke et al. 2010). It is important to note that the sexuals in the nests we collected were still young and their δ^15^N values represented their recent trophic past. The fact that δ^15^N values did not differ significantly between adult workers and pupal workers reveals that no notable shift in the diet took place over the workers' lifespan (several months). However, the higher δ^15^N values of queens compared to those of females may indicate that the former continue feeding on a more protein-rich diet throughout their life. It might be argued that the greater need for protein is related to egg laying and vitellin production.

Differences in ground temperature stemming from fire-induced changes in vegetative cover could also have had a significant impact on the reproductive output of colonies. However, until now, increases in nest temperature have only been reported to augment female production in some ant species (Rosengren and Pamilo 1986; Aron et al. 1994; Tillberg et al. 2006; *Menke* et al. 2010). In the Argentine ant, laboratory work has shown that eggs laid at lower temperatures are not fertilized and that they develop into males (Aron et al. 1994). In *Formica exsecta*, queen numbers were shown to increase in nests receiving more solar radiation (Kümmerli and Keller 2008). Distinguishing between the effects of resource availability and temperature may be relatively complicated in nature because the vegetation often mediates both microclimatic conditions (e.g., reduced temperature via shade) and resource availability. Further experiments under laboratory-controlled conditions may test the role of temperature or/and resource availability on primary and secondary sex ratios.

In conclusion, although the fire resulted in important changes in the plant and arthropod communities, we did not find much evidence of its impact on ant trophic interactions. In contrast to the general assumption that ants are trophically flexible, our work shows that they maintained the same diet in spite of a significant habitat disturbance. Our focal species, *A. gibbosa*, was resilient to fire and its worker abundance was the same in burned and unburned plots. However, fire had a major effect on sex allocation, which may have important consequences at the population level in the longer term. If colonies fail to produce females over one or maybe more seasons, it would be expected to provoke reduced colony recruitment in burned areas. We encourage more studies investigating resource and sex allocation in ant assemblages. We also encourage studies focusing on longer term population genetics in order to detect potential genetic bottlenecks and founder effects provoked by habitat disturbance.

## Supporting Information

**Figure S1.** Biotic and abiotic conditions in burned and unburned zones. Mean (±SE) of ground surface temperature and relative humidity during the month of July 2010 (burned in black and unburned in white). Photographs correspond to the burned and unburned zones containing the study plots (see Material and Methods).

**Figure S2.** Characterisation of the plant community in burned (*BS*) and unburned (*UBS*) plots: Percentage of species abundance (plot means ± SE, *n* = 5).

**Figure S3.** Invertebrate abundance, biomass, and the Shannon index of diversity H (plots means ± SE, *n* = 5) across burned (black bars, *BS*) and unburned (white bars, *UBS*) plots. Abundance is based upon the mean number of individuals of each group collected within each plot.

**Table S1.** Effect of fire on granulometry and soil chemistry (mean [±SE]). *F*-values come from ANOVA tests (*P* < 0.05). Soil chemistry did not differ significantly between burned and unburned plots.
