# Supplementary material for: The effects of fire on ant trophic assemblage and sex allocation
Source: Ecol Evol. 2013 Dec 6;4(1):35–49. doi: 10.1002/ece3.714 (PMC3894886; doi:10.1002/ece3.714)
Supplement: Supplementary file 1 [file ece30004-0035-SD1.docx]

**Supporting Information**

**- Caut et al. The effects of fire on ant trophic assemblage and sex allocation**

**Figure S1.** Biotic and abiotic conditions in burned and unburned zones. Mean (±SE) of ground surface temperature and relative humidity during the month of July 2010 (burned in black and unburned in white). Photographs correspond to the burned and unburned zones containing the study plots (see Materials and Methods).


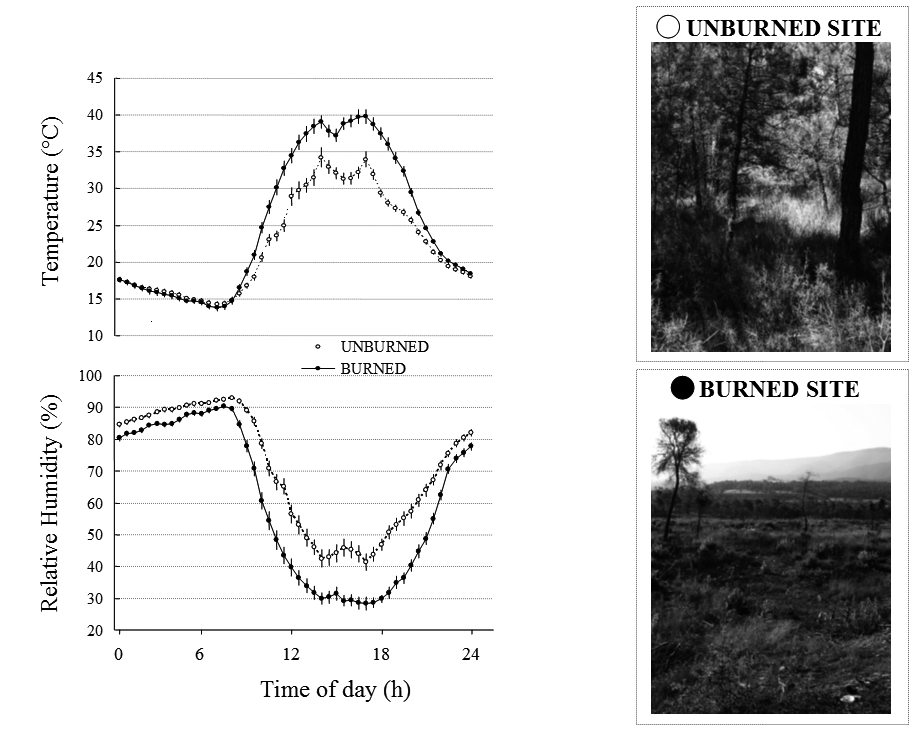


**Supporting Information - Caut et al. The effects of fire on ant trophic assemblage and sex allocation**

**Table S1.** Effect of fire on granulometry and soil chemistry (mean (±SE)). F-values come from ANOVA tests (P < 0.05). Soil chemistry did not differ significantly between burned and unburned plots.

|  |  |  |  |  |  |  |  |
| --- | --- | --- | --- | --- | --- | --- | --- |
|  |  |  |  |  |  |  |  |
| Parameters |  | Burned |  | Unburned |  | F_1,18_ | P |
|  |  |  |  |  |  |  |  |
| Fine sand (%) |  | 8.10 (1.47) |  | 13.42 (0.76) |  |  |  |
| Coarse sand (%) |  | 49.52 (10.94) |  | 25.24 (1.40) |  |  |  |
| Silt (%) |  | 27.24 (8.71) |  | 41.40 (1.69) |  |  |  |
| Clay (%) |  | 15.12 (2.12) |  | 19.90 (0.60) |  |  |  |
|  |  |  |  |  |  |  |  |
| pH (Ext 1:2.5) |  | 7.88 (0.12) |  | 8.02 (0.07) |  | 1.08 | 0.313 |
| Phosphorus Bray-Kurtz (mg/kg) |  | 1.59 (0.53) |  | 1.47 (0.29) |  | 0.04 | 0.844 |
| Assimilated potassium (mg/kg) |  | 141.80 (18.35) |  | 128.90 (7.02) |  | 0.43 | 0.520 |
| Oxidisable organic carbon (%) |  | 4.10 (0.40) |  | 4.28 (0.33) |  | 0.11 | 0.743 |
| Oxidisable organic material (%) |  | 7.07 (0.69) |  | 7.37 (0.56) |  | 0.12 | 0.737 |
| N Kjeldahl (%) |  | 0.24 (0.02) |  | 0.29 (0.02) |  | 1.66 | 0.213 |
|  |  |  |  |  |  |  |  |
|  |  |  |  |  |  |  |  |

**Supporting Information - Caut et al. The effects of fire on ant trophic assemblage and sex allocation**

**Figure S2.** Characterisation of the plant community in burned (*BS*) and unburned (*UBS*) plots: Percentage of species abundance (plot means ± SE, n = 5).

**Supporting Information - Caut et al. The effects of fire on ant trophic assemblage and sex allocation**

**Figure S3.** Invertebrate abundance, biomass, and the Shannon index of diversity H (plots means ± SE, n = 5) across burned (black bars, *BS*) and unburned (white bars, *UBS*) plots. Abundance is based upon the mean number of individuals of each group collected within each plot.
